# Workshop proceedings: GWAS summary statistics standards and sharing

**DOI:** 10.1016/j.xgen.2021.100004

**Published:** 2021-10-13

**Authors:** Jacqueline A.L. MacArthur, Annalisa Buniello, Laura W. Harris, James Hayhurst, Aoife McMahon, Elliot Sollis, Maria Cerezo, Peggy Hall, Elizabeth Lewis, Patricia L. Whetzel, Orli G. Bahcall, Inês Barroso, Robert J. Carroll, Michael Inouye, Teri A. Manolio, Stephen S. Rich, Lucia A. Hindorff, Ken Wiley, Helen Parkinson

**Affiliations:** 1 European Molecular Biology Laboratory, European Bioinformatics Institute, Wellcome Genome Campus, Hinxton, UK; 2 BHF Data Science Centre, Health Data Research UK, London, UK; 3 Division of Genomic Medicine, National Human Genome Research Institute, National Institutes of Health, Bethesda, MD 20892, USA; 4 Cell Genomics, Cell Press, 50 Hampshire St., 5th Floor, Cambridge, MA 02139, USA; 5 Exeter Centre of Excellence in Diabetes (EXCEED), University of Exeter Medical School, Exeter, UK; 6 Department of Biomedical Informatics, Vanderbilt University Medical Center, Nashville, TN, USA; 7 Cambridge Baker Systems Genomics Initiative, Department of Public Health and Primary Care, University of Cambridge, Cambridge CB1 8RN, UK; 8 Cambridge Baker Systems Genomics Initiative, Baker Heart and Diabetes Institute, 75 Commercial Rd., Melbourne 3004, VIC, Australia; 9 The Alan Turing Institute, London, UK; 10 Center for Public Health Genomics, University of Virginia, Charlottesville, VA 22908, USA

## Abstract

Genome-wide association studies (GWASs) have enabled robust mapping of complex traits in humans. The open sharing of GWAS summary statistics (SumStats) is essential in facilitating the larger meta-analyses needed for increased power in resolving the genetic basis of disease. However, most GWAS SumStats are not readily accessible because of limited sharing and a lack of defined standards. With the aim of increasing the availability, quality, and utility of GWAS SumStats, the National Human Genome Research Institute-European Bioinformatics Institute (NHGRI-EBI) GWAS Catalog organized a community workshop to address the standards, infrastructure, and incentives required to promote and enable sharing. We evaluated the barriers to SumStats sharing, both technological and sociological, and developed an action plan to address those challenges and ensure that SumStats and study metadata are findable, accessible, interoperable, and reusable (FAIR). We encourage early deposition of datasets in the GWAS Catalog as the recognized central repository. We recommend standard requirements for reporting elements and formats for SumStats and accompanying metadata as guidelines for community standards and a basis for submission to the GWAS Catalog. Finally, we provide recommendations to enable, promote, and incentivize broader data sharing, standards and FAIRness in order to advance genomic medicine.

## Teaser

MacArthur et al. report on a GWAS Catalog community workshop on the sharing of summary statistics to ensure that these are findable, accessible, interoperable, and reusable (FAIR). They provide recommendations and guidance to incentivize broader data sharing, standards, and FAIRness in order to advance genomic medicine.

## Introduction

Genome-wide association studies (GWASs) have brought enormous progress in mapping the genetic basis of common diseases or traits,^1^^,^^2^ where genetic predisposition is shared across thousands of mostly common variants with individually modest effects on population risk. Since 2005,^3^ GWASs have successfully identified thousands of genomic regions significantly associated with common diseases, with notable successes in type 2 diabetes (T2D)^4^ and coronary artery disease.^5^ This approach was successfully applied at the start of the coronavirus disease (COVID) global pandemic in 2020, with newly established international collaborations driving COVID-19 GWASs and making all data publicly available.^6^ GWAS datasets are increasingly publicly shared, and these datasets are widely used to further basic research, as well as translation, including in drug-discovery pipelines.^7^

The number of published GWASs has continually increased, with 265 new publications in the first 6 months of 2021 compared with 209 in the same period of 2019. In addition, the complexity and scale of the data grow. This includes the interrogation of larger sample sizes, driven by prospective cohorts and biobanks. Studies also increasingly include a broader range of data types in a single publication, with deep phenotyping or health information, including newer -omic phenotypes (e.g., lipidomic, proteomic, metabolomic, etc.).^8^, ^9^, ^10^ Recent publications have included GWASs of ∼4,000 brain-imaging traits,^11^ ∼1,500 protein biomarkers,^12^ and 778 traits in the UK Biobank (UKBB).^13^ Dense imputation panels have increased the number of variants analyzed, with a typical GWAS now including more than 8 million variants. GWAS analytical methods are also beginning to be applied to whole-genome sequencing data,^14^ with the potential for vastly increased coverage of the genome and inclusion of rare variants.

The GWAS Catalog^15^ was established with the aim of providing a central repository for variant-trait associations identified through GWASs, serving as a starting point for investigations to identify causal variants, understand disease mechanisms, and establish targets for new therapies. GWAS datasets are both submitted by the research community and identified via the peer-reviewed literature and then curated and annotated according to transparent standards by GWAS Catalog curators and made available via a user-friendly web-based search interface. As of June 2021, the Catalog contains more than 5,000 publications containing more than 20,000 individual GWASs, with more than 250,000 top associations (p < 1 × 10^−5^). Downloadable flat files and a representational state transfer application programming interface (REST API) provide flexible access to the data. Data from the GWAS Catalog are openly shared and re-usable, which has enabled integration into numerous other reference databases, such as Ensembl and the Open Targets resources.

GWAS SumStats are defined as the aggregate p values and association data for every variant analyzed. Public sharing of GWAS SumStats received support through an update to the National Human Genome Research Institute (NHGRI) genomic data-sharing policy in 2018 (web resources). The utility of GWAS datasets has been vastly increased by SumStats sharing, enabling broader meta-analyses and optimization for diverse ancestry, in addition to trait pleiotropy, phenome scans, polygenic risk prediction, and Mendelian randomization.^16^^,^^17^

As a response to the community need for SumStats, in 2018, the GWAS Catalog began identifying SumStats made publicly and freely available by authors at other locations and hosting those in the Catalog. Direct submissions from authors have also been accepted since June 2020. SumStats are easily findable in the GWAS Catalog search interface, according to publication, trait, or other search terms. Files are downloadable from the ftp site in our standard format^15^ and also via a dedicated API. All data are made available freely and without restriction or registration requirements, in contrast to the controlled access offered by related resources, such as the Database of Genotypes and Phenotypes (dbGaP).

The GWAS Catalog has seen a sharp increase in bothsharing of GWAS SumStats and their downloads in the past 2–3 years. SumStats are available for 22% of publications published in 2020 and represented in the GWAS Catalog, compared with 9% of Catalog publications overall. GWAS Catalog SumStats downloads were 3-fold greater in the first 6 months of 2020 compared with the same period in 2019. Despite that shift, most GWAS publications continue to not make their SumStats publicly available, either in the GWAS Catalog or elsewhere.^18^ The reasons given for limited sharing include technical challenges, concerns regarding data misuse, privacy concerns,^19^ and the perceived lack of an appropriate repository. In addition, for those that do share GWAS SumStats, they are often not submitted to a centralized repository and are instead made available only on dispersed project-centric websites, presented in a range of different formats, and largely lacking rich, searchable metadata. The lack of a centralized repository and global standards for data content and format presents challenges for users who must find, harmonize, organize, and manage the data before analyses.

We convened a community workshop to address the standards, infrastructure, and incentives required to promote and enable the sharing of SumStats. During the workshop, we evaluated the barriers to SumStats sharing, both technological and sociological, and developed an action plan to address these challenges as follows:

1. Ensuring SumStats and study metadata are findable, accessible, interoperable, and reusable (FAIR)^20^ and relevant to the user community
2. Establishing a community standard for reporting GWAS SumStats and metadata
3. Identifying strategies to incentivize sharing of SumStats

Here, we review the GWAS SumStats standards and sharing workshop proceedings and community discussions. We report our recommendations and planned implementations to realize the broad sharing of GWAS SumStats and to ensure their FAIRness. Our recommendations include timely deposition of datasets in the GWAS Catalog, as the recognized central repository, and standards for reporting elements and formats.

## Workshop organization

**Figure 1:**
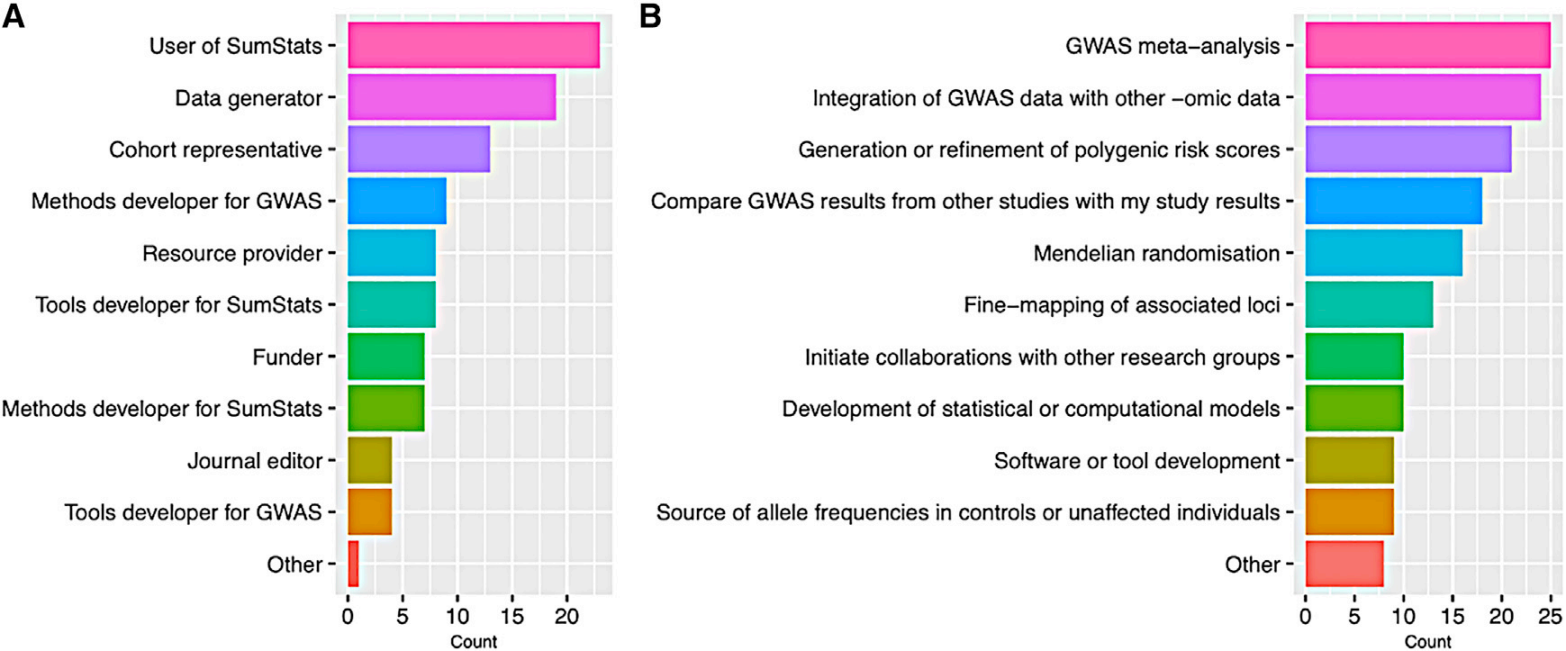
Workshop attendees (A and B) Breakdown of workshop attendees by stakeholder category (A) and planned uses of GWAS SumStats (B) for 37 workshop attendees (35 for planned uses) who completed the pre-workshop survey. Attendees were able to select multiple stakeholder categories and planned uses.

To ensure broad input from the scientific community, workshop participants were selected to represent the diversity of the stakeholder space and SumStats users (Figure 1). Session chairs were invited based on their expertise and interests aligned with the workshop aims. The objectives of each session were agreed upon among session chairs, the GWAS Catalog, and NHGRI program directors. Ahead of the workshop, an online survey was shared with attendees to assess community needs and opinions regarding blockers to data sharing and SumStats content accessibility, infrastructure, and incentivization.

The workshop was held via webinar on June 1 and 2, 2020. Roughly 50 attendees took part each day. Teri Manolio opened the workshop with a keynote on the history and future of the GWAS Catalog. The rest of the workshop was organized around six topic sessions: data content, FAIR, incentivization of sharing, infrastructure requirements, data update cycle, and forward look. Survey results were presented in the relevant sessions by a member of the GWAS Catalog team to drive discussion and facilitate decision making. The pre-workshop briefing document, full survey results, agenda, attendees, and session videos are available on the GWAS Catalog website (https://www.ebi.ac.uk/gwas/docs/sharing-standards-workshop).

## Workshop proceedings

### Data content

This session, chaired by Inês Barroso, aimed to determine the requirements for GWAS SumStats data content and format, considering the needs of stakeholders. We agreed that data content and format requirements must be known in advance of data collection (and ideally study onset) to ensure that the data are available and have consent for sharing. These requirements should be designed to maximize content and usefulness, to minimize the burden for the data supplier, and to account for the lack of feasibility in obtaining data for certain study types. They should also be sensitive to privacy concerns, diverse users, and study participant concerns. The format should be flexible to contain single or multiple GWAS SumStats in the same file. There were differing opinions on the preferred file format (flat file or variant call format [VCF])^21^ for SumStats, which depends on use case and stakeholder needs (see Box 1, Workshop recommendations 8, working group on “Data content and format”).

Box 1 **Workshop recommendations on sharing of GWAS summary statistics**

We recommend these actions to enable broader sharing of GWAS SumStats and to ensure that SumStats and study metadata are FAIR. These recommendations were compiled by the organizers and session chairs, with feedback gathered during the workshop and the wider community in the pre-workshop survey.

1. Establish a comprehensive, central resource of GWAS SumStats

We recommend establishing a comprehensive and sustainable resource for all GWASs and propose that the GWAS Catalog be recognized as the central resource for all human GWASs.

2. Submit all GWAS SumStats to the GWAS Catalog

GWAS SumStats and supporting metadata should be submitted to the GWAS Catalog at the time of submission of a manuscript to a journal and/or a preprint server. Accession IDs for GWAS SumStats should be cited in the relevant manuscript and any other relevant material.

3. Promote or require submission to the GWAS Catalog

We call on journal editors, funders, and cohort representatives to promote or require early submission to the GWAS Catalog, pointing authors to the GWAS Catalog and expecting submission before journal submission (journal editors) or as a requirement for sample use (cohort representatives) or funding (funders).

4. Ensure GWAS SumStats and metadata meet FAIR indicators

GWAS SumStats should be made available following the FAIR indicators (Table 2). These FAIR indicators will be adopted by the GWAS Catalog.

5. Adopt a standard format and elements for GWAS SumStats

GWAS SumStats should include these standard elements: variant ID or chromosome plus base pair location, p value, effect allele, other allele, effect allele frequency, effects (odds ratio or beta), and standard error (Table 1).

6. Data should be versioned and linked to relevant resources

GWAS SumStats and accompanying metadata should be versioned to enable users to identify the most recent dataset. The GWAS Catalog will develop a data update and versioning strategy to meet those needs. Linking from GWAS SumStats and metadata to relevant datasets in other databases (e.g., dbGaP, EGA, BioData Catalyst, and AnVIL) should be improved. The GWAS Catalog will develop improved cross-linking to relevant databases.

**Areas for further discussion:**

7. Diversity and privacy

To ensure the Catalog can meet the needs of all studies, including those with more-sensitive datasets or alternative study designs, we will convene working groups to gather additional evidence and identify additional functionality required. We recommend that different data-sharing requirements be considered for datasets determined to be sensitive, where required for privacy or regulatory reasons. We are convening a working group to provide guidance on communicating and mitigating the risks associated with sharing of SumStats (“Diversity and privacy” working group).

8. Data content and format

To further assess and finalize metadata content, variant identification, and file format requirements, including for association testing with multiple variants in a region, we are convening a working group (“Data content and format” working group).

We agreed to an initial set of standard reporting elements for GWAS SumStats (Table 1) based on the results of the pre-workshop survey (web resources) and workshop discussion. The mandatory reporting elements should include p value and variant ID or genomic location (plus genome build),^15^ effect allele, other allele, effect size (odds ratio or beta), and standard error (Table 1; Box 1, Workshop recommendations 5). Alternative ways of representing variants were discussed because it is recognized that using reference SNP IDs (rsIDs) or genomic location does not facilitate unambiguous identification of all variants. Attendees also suggested that variant representation should be compliant with Global Alliance for Genomics and Health (GA4GH) standards (https://vrs.ga4gh.org/en/stable/) and be able to represent haplotypes. It was also noted that the standard should specify the level of detail required for each value, for example, the number of significant digits.

**Table 1:** Recommended standard reporting elements for GWAS SumStats

Data element	Column header	Mandatory/Optional
variant id	variant_id	One form of variant ID is mandatory, either rsID or chromosome, base pair location, and genome build^a^
chromosome	chromosome	
base pair location	base_pair_location	
p value	p_value	Mandatory
effect allele	effect_allele	Mandatory
other allele	other_allele	Mandatory
effect allele frequency	effect_allele_frequency	Mandatory
effect (odds ratio or beta)	odds_ratio or beta	Mandatory
standard error	standard_error	Mandatory
upper confidence interval	ci_upper	Optional
lower confidence interval	ci_lower	Optional

Data elements have been recommended as mandatory if >50% of pre-workshop survey respondents indicated that preference.

^a^We agreed that other variant ID formats should be supported. Implementation of those standards will be addressed by the working group “Data Content and Format.”

Although the sharing of SumStats poses low risk to participants’ privacy, there could be a small risk of identifying individual level data, and those risks are greater for certain studies, such as those that include individuals from isolated populations or with rare traits. We agreed that it was important to acknowledge the potential for risk by specific study criteria and to provide guidance on how to minimize risk. It was suggested that the requirements for data sharing could be different for studies that have determined sensitive datasets: for example, the risk of identification could be reduced by not requiring the sharing of study-specific minor allele frequencies (MAFs) or reducing the decimal points required for p values.

### FAIR

During this session, chaired by Robert Carroll, we identified FAIR indicators that can be used to assess whether GWAS data conform to the FAIR Guiding Principles,^20^ taking into account the needs of users (Table 2). We discussed which of those indicators are already being met and where improvements are required.

**Table 2:** FAIR indicators

Core FAIR principle	FAIR principle	FAIR indicator
Findable	F1. (meta)data are assigned a globally unique and persistent identifier	Each GWAS is assigned a unique identifier that can be resolved externally through IDENTIFIERS.org e.g., GCST no.
	F2. data are described with rich metadata (defined by R1 below)	Each GWAS is described by the metadata elements listed in “Proposed metadata standard reporting elements”^a^
	F3. metadata clearly and explicitly include the identifier of the data it describes	Metadata include the accession ID and are linked to the GWAS SumStats they describe
	F4. (meta)data are registered or indexed in a searchable resource	GWAS is searchable in the GWAS Catalog by accession ID, trait, publication, author, or locus (variant, gene, cytogenetic, or chr:bp-bp region)
Accessible	A1. (meta)data are retrievable by their identifier using a standardized communications protocol	Metadata can be easily viewed on the GWAS Catalog web interface, with a specific page for each GWAS, accessible through a stable URL, which includes the accession ID, with a download link for the SumStats Metadata that can be retrieved from the GWAS Catalog’s REST API (https://www.ebi.ac.uk/gwas/docs/api) using the accession ID
	A1.1 the protocol is open, free, and universally implementable	The GWAS Catalog (https://www.ebi.ac.uk/gwas/) website and datasets are freely accessible to all
	A1.2 the protocol allows for an authentication and authorization procedure, where necessary	Not applicable
	A2. metadata are accessible, even when the data are no longer available	Metadata will remain accessible via the accession ID, even if the SumStats are no longer available. Archived versions of GWAS Catalog metadata are available
Interoperable	I1. (meta)data use a formal, accessible, shared, and broadly applicable language for knowledge representation	Metadata are accessible from the GWAS Catalog REST API (https://www.ebi.ac.uk/gwas/docs/api) using JSON formats
	I2: (Meta)data use vocabularies that follow the FAIR principles	Traits are represented using Experimental Factor Ontology^22^ terms, ancestry information is represented using the GWAS Catalog’s standardized ancestry framework,^23^ and all variants (dbSNP accession ID), genes (HGNC symbols), and chromosome locations (Genome Reference Consortium genome assembly GRCh38) use accepted standards, vocabularies, and naming conventions
	I3. (meta)data include qualified references to other (meta)data	Links are provided to relevant external data, e.g., Europe PMC, and to relevant GWAS Catalog data, e.g., the trait and publication pages
Reusable	R1. (meta)data are richly described with a plurality of accurate and relevant attributes	Each GWAS is described by the metadata elements listed in “Proposed metadata standard reporting elements”^a^
	R1.1. (meta)data are released with a clear and accessible data usage license	All GWAS Catalog data are made available through EMBL-EBI’s standard terms of use (https://www.ebi.ac.uk/about/terms-of-use/), and submitted summary statistics are additionally made available under the terms of CC0 (https://creativecommons.org/publicdomain/zero/1.0/)
	R1.2. (meta)data are associated with detailed provenance	Each GWAS is linked to a source publication that can be accessed by either a digital object identifier (DOI) or ID (PMID)
	R1.3. (meta)data meet domain relevant community standards	Metadata and SumStats are made available using the standards agreed to in this workshop^a^

Our recommended FAIR indicators for GWAS SumStats. We list each core FAIR principle and the associated indicators and provide examples of how they are implemented in the GWAS Catalog.

a This indicator is not currently met in full by the GWAS Catalog. The data standards agreed to in this workshop require extensions or modifications to GWAS Catalog data content or formats, which we plan to implement soon.

We agreed that unique and persistent accession IDs must be provided for SumStats at the point of submission of the dataset to a database and prior to publication of the study in a journal. This allows journals to check that the dataset is accessible and for the inclusion of accession IDs in the publication. For the reporting of SumStats, most attendees agreed that the following metadata elements should be mandatory: sample size (including number of cases/controls), sample ancestry, imputation method and reference panel, covariates, trait measurement (e.g., self-reported versus clinically diagnosed), sample inclusions/exclusions, additional cohort descriptors (e.g., cohort names), analysis plan (e.g., model and software used), genotyping/sequencing technology, minor allele frequency cutoff, trait quality control, and number of variants analyzed. Attendees discussed that there is an incentive to meet only the minimum requirements; therefore, those requirements should include all useful information; otherwise, those data may not be shared. There were differing opinions on the preferred metadata format, either incorporated within the SumStats file or in a separate file, and that format needs further discussion. However, it was agreed that representing metadata using a standard file can be challenging, and tools to support users in that would be extremely beneficial.

License restrictions and lack of transparency regarding what uses are restricted can be significant barriers to data sharing and reusability. The GWAS Catalog analyzed publications from 2019 and 2020 in which the summary statistics were not shared restriction-free via the Catalog because of some form of research or license restriction. Many of those restrictions are participant or cohort centric and reflect an attempt to protect research participants, for example, restrictions on attempting to identify participants, research that may lead to stigmatizing individuals or groups, or the use of data for commercial purposes. Attendees agreed that it would be useful to have a “recommended license,” which would enable reuse but protect research participants (see Box 1, Workshop recommendations 7, “Diversity and privacy” working group). On the other hand, some data generators imposed investigator-centric restrictions that inherently limit reuse, for example, by prohibiting redistribution. Ways to overcome barriers for data generators who are reluctant to share without such restrictions are discussed in more detail in the Incentivization of sharing session section below.

We also agreed that improved linking among databases is required, for example, linking among different datasets hosted in different repositories for the same cohort or sample set (see Box 1, Workshop recommendations 6).

### Incentivization of sharing

The aim of this session, chaired by Orli Bahcall, was to identify barriers to sharing of GWAS data and define strategies to overcome those barriers, including identifying incentives for data sharing. From her experience in working on the development of data-sharing programs and with a broad range of GWAS producers, she proposed that the barriers to sharing and the strategies required to overcome them differ among GWAS producers who want to share the dataset but meet challenges and those who are reluctant to share from the outset.

Most of the challenges faced by GWAS producers who are amenable to data sharing can be reduced or eliminated by the presence of a suitable repository that supports a submitter’s needs: ease of submission, short waiting times, clear requirements, provisioning of an accession identifier at time of submission, support for versioning, ability to submit the dataset early (soon after generation and before posting a first preprint manuscript reporting the dataset), optional access control, and setting embargoes.

For the “reluctant” data sharers, reasons may relate largely to either understanding of data sharing or a culture of ownership and competition. First, some may be deterred by overestimation of the minimal risks associated with sharing of SumStats.^19^^,^^24^ In relation to privacy and de-identification issues, however, those barriers have been addressed by the 2018 NIH statement (see web resources). Compounding that issue are concerns over consent and regulatory requirements; there may be a lack of either transparency or clarity on whether participants’ consent agreements allow for sharing of SumStats. Second, even though genomics has paved the way in data sharing for biological sciences and is the most progressive community in commitment to open science, a widespread culture of data ownership continues. These data producers maintain private or restricted ownership of their data in the interest of competitive advantage for publications and other research outputs.

Sharing in the “reluctant” group can often be increased by providing clear guidance on the community needs and the benefits from sharing, the minimal privacy risks involved, and current guidelines (web resources). Providing personalized guidance on sharing for sensitive datasets is also beneficial, advising on how risks to participants in these studies should be minimized; for example, through controlled access or limits to information included in public sharing (see Box 1, Workshop recommendations 7. “Diversity and privacy” working group).

Regulations applied by either journals or funders (NIH grant; web resources) are among the common incentives for sharing other types of data. To be most effective, these regulations must require the SumStats to be deposited soon after generation or, at the latest, before a manuscript publication in a journal (Box 1, Workshop recommendations 2 and 3). Until recently, a barrier to this has been the lack of an appropriate repository that accepts submissions pre-publication; however, the GWAS Catalog now supports this and issues accession IDs at submission. This advance has allowed *Cell Genomics* to require deposition in the GWAS Catalog with the first manuscript submission to the journal since May 2020 as a condition of consideration for review, so that the datasets and access can be reviewed during the peer review process.^25^

### Infrastructure requirements

Infrastructure for data management and storage is essential to enable sharing of GWAS data and to support data deposition, hosting, and distribution. In this session, chaired by Mike Inouye, we aimed to evaluate stakeholder infrastructure and data hosting requirements.

Workshop participants recommended a centralized repository or aggregator of GWAS data (Box 1, Workshop recommendations 1), supporting ease of data findability, accessibility, standardization, and data transfer to downstream tools (e.g., LD Hub^26^ and MR-Base).^27^ In this model, the repository serves as the intermediary, supporting submission by data generators and access by data users. This presents the question of where the burden of formatting data should be placed. Most participants felt that this burden should lie with submitters, who can facilitate the validation of submitted data to support harmonization and downstream uses. To mitigate that burden, formatting and validation tools for submitters are essential, along with support for submission of large volumes of data, accession IDs being provided upon submission, versioning support, and protocols that are free to use. For users of SumStats, the most important requirement is access to harmonized data. This should be supported by flexibility in access methods, including filtering across SumStats, robust APIs, and dataset download.

Although there are many advantages to a centralized resource, particularly for users of the data, some studies may prefer or require local control of datasets because of concerns regarding privacy or misuse. However, controlled access within a centralized resource may also meet those data management needs. We will further consider the best way to support those needs and to ensure the data are findable and accessible for approved uses (Box 1, Workshop recommendations 7, “Diversity and privacy” working group).

### Data update cycle

In this session, chaired by Raymond Walters, we discussed the requirements for the GWAS SumStats data update cycle, including when datasets should be submitted and how to handle updates and versioning.

We agreed that a priority for a repository should be handling submissions of studies that are near publication in a journal, including manuscripts posted as preprints and should include the provision of accession IDs, so that these can be included in the journal publication (Box 1, Workshop recommendations 2). In addition, sharing of data from GWASs that are not associated with a preprint or journal publication (e.g., the UK Biobank analyses made available by the Neale lab http://www.nealelab.is/) is also increasingly important.

From our general observations, most SumStats were not seen to change substantially between generation and publication of the study in a journal. However, over that time, there may be an initial release and then several versions updating the SumStats. Submitters of those datasets need support for versioning and the addition of metadata annotation. Versioning needs to allow users to identify and access the most recent dataset (SumStats and supporting metadata) (Box 1, Workshop recommendations 6). Submitters also need to be able to retract data, where necessary. After retraction, the accession ID and Uniform Resource Identifier (URI) should be maintained with an indication of the data retraction.

Workshop participants also discussed sharing of partial SumStats because of restrictions imposed by cohorts or data controllers. Although workshop attendees expressed concern at cohorts imposing restrictions on data sharing, it was agreed that partial sharing should be accepted, where full sharing is not possible for regulatory reasons. These SumStats should be flagged as partial, in addition to versioning, to make users aware and to encourage submission of a full version. The concern raised in allowing partial sharing is that some will use this to avoid full sharing without legitimate reasons; it is hoped that defining sharing recommendations (Box 1, Workshop recommendations 2 and 7), including guidance on risks and how these should be mitigated, will empower cohort leaders, funders, and journal editors to apply regulations (see Incentivization of sharing).

### Forward look

In this session, chaired by Stephen Rich, we considered requirements for alternative GWAS design and emerging technologies.

To ensure that data from alternative GWAS designs are interpretable, it is important that format and content sharing requirements (for both SumStats and metadata) take into account different study designs and technologies (Box 1, Workshop recommendations, working group 1. “File format and content”). For most GWASs focused on testing association with single variants, SumStats data elements will be comparable, with differences in study design captured as metadata. However, GWAS testing association with multiple variants in a gene/region (using burden/SKAT-O tests) or SNP-by-SNP interactions will require that additional information be specified in the SumStats standard. It is also important to specify minimally acceptable responses to each of the required data items, for example, by defining structured data elements made available through multiple choice or defining the minimum number of decimal places that should be supplied.

Whole-genome and whole-exome sequencing now represents a viable alternative to array-based genotyping for use in GWASs. To overcome issues of reduced power associated with multiple testing of rare variants identified through sequencing, statistical methods have been developed to evaluate aggregate association with multiple genetic variants in a region (e.g., gene). In a pilot study^14^ of GWAS Catalog data from 167 publications, we found that the reporting of aggregate association results is extremely variable, with minimal information included as standard in shared SumStats, often only locus ID and p value. Workshop attendees agreed that there is a need to standardize the reporting of those aggregate-association tests, both in how the tests are performed and also for the results, including the set of variants that contribute to each test. We agreed that standard reporting guidelines need to be defined for SumStats and metadata from GWAS testing association with multiple variants in a region. We will review further to establish a definitive list of required elements and standard format, as part of the “Data content and format” working group (Box 1, Workshop recommendation 8).

## Outlook

Here, we report our recommendations to realize the broad sharing of GWAS SumStats and to ensure FAIRness. Based on our analyses, community survey, and workshop, we have resolved primary recommendations to enable the sharing of SumStats (Box 1). Our recommendations for community adoption include timely deposition of datasets in the GWAS Catalog and standards for reporting elements and formats. We are continuing discussions in our working groups to explore and resolve outstanding issues and to develop additional recommendations. We hope that this collective work will enable broad data sharing not only for GWAS summary statistics but also to feed into other ongoing efforts to standardize and share data,^28^ with the ultimate goal of advancing the field of genomic medicine.

## Web resources

The NHGRI-EBI Catalog of human genome-wide association studies (GWAS Catalog), https://www.ebi.ac.uk/gwas
The GWAS summary statistics standards and sharing workshop, including pre-workshop briefing document, full survey results, agenda, attendees, and session videos, https://www.ebi.ac.uk/gwas/docs/sharing-standards-workshop
The GWAS Catalog’s summary statistics submission interface, https://www.ebi.ac.uk/gwas/deposition
NIH Genomic Data Sharing Policy, https://grants.nih.gov/grants/guide/notice-files/NOT-OD-19-023.html
NIH grant to promote data sharing in cancer epidemiology studies, https://grants.nih.gov/grants/guide/pa-files/PA-18-748.html
GA4GH Variation Representation Specification, https://vrs.ga4gh.org/en/1.0/#:∼:text=The%20Variation%20Representation%20Specification%20(VR,improve%20sharing%20of%20genetic%20information.
Ensembl, https://www.ensembl.org
Open Targets, https://www.opentargets.org
The International Common Disease Alliance, https://www.icda.bio/
